# Cause of Yellow Fever

**Published:** 1857-02

**Authors:** 


*Cause of Yellow Fever.*

The following extracts from the published proceedings of the

New York Academy of Medicine, contain a summary of the views of Dr. E. H. Barton, of New Orleans, on this interesting subject. Dr. Barton has had the most ample opportunities for observations concerning the cause or causes which originate and propagate the Yellow Fever, and has improved them laboriously and carefully. His opinions are, therefore, entitled to much consideration.

'*Cause of Yellow Fever.*—There are two conditions necessary to the creation of yellow fever: an elevated temperature and a high dew-point, form the blades of the "shears of fate," united by miasm and filth. The report of the sanitary commission had stated this fact, and that whenever the dew-point fell to 60°, the fever ceased invariably. The experience of the subsequent years had corroborated this view, both in New Orleans, Savannah, Charleston, &c.

'The paper which he should read to the Academy was prepared for New Orleans, but it contained some statements, intended for this Academy, in opposition to the views expressed here within the last year.

'*First*—He should make some *corrections* where he had been misrepresented. He had never said that disturbance of the soil alone would produce yellow fever.

'*Second*—He had never stated that a high dew-point was the cause of yellow fever: but that it was the conjunction of these two elements with filth in certain proportions that was the cause. The three elements were necessary.

'In regard to the report of the commission, which had generally been well received by the profession, there was skepticism on two points, the influence of a high dew-point and the necessity of a specific cause, and these points he proceeded to explain. Here he took notice of statements made a year since to the Academy, by Dr. Stone, of New Orleans, whose high position rendered it necessary that these views should be met.

'The condition of the atmosphere was very important. He had frequently known unacclimated persons, visiting an infected district, to be attacked in two hours when the dew-point was high.

'I predicted the yellow fever of 1853, in May. The spring was dry, the rainy season being but seven days and seven nights, but in June and August there was much rain; for thirty days and nights it rained, and the dew-point averaged 70 71-100. Yellow fever depends on meteorological changes, and as the dew-point rises and falls this disease varies. A few cases may occur sporadically, but there never will be an epidemic.

'Dr. S. said that this disease had no relation with intermittents, and that it was more likely to attack localities where miasmatic diseases prevailed. It is charity to suppose that the author has been misreported in this particular, the fact being so directly opposed to this view.

'He wished to correct one very prevalent idea, that drouth and dryness are synonymous. A hydrometer is the only test of this atmospheric condition. Rapid rains deplete the air and leave it dryer than before. Sandy soils absorb it, while clayey ones retain it, and the air is, consequently, correspondingly more or less dry. It is well known that foggy weather is generally without rain, yet no one would pretend that the air was not laden with moisture. Humidity is a necessary constituent in many diseases, as cholera, cholera infantum, sun-stroke, &c.

'Wherever yellow fever occurs in any place for the first time, it is always accompanied by marked atmospheric changes; the changes are noted at its departure, and these show how futile are the terms indigenous, imported, and the like. A few cases may occur after a frost, but there is no epidemic. So we see cholera occurring in cold weather, and even in Russia, where the thermometer is near zero; but it is forgotten that this is the out-door temperature. The in-door temperature, where the disease actually occurs, is about 80, or summer heat. The seasons when yellow fever is rife in New Orleans, are peculiarly damp. The city is always damp. Goods spoil kept in our stores one year, and flour frequently in a few hours; for notwithstanding the peculiar dampness of our city little or no efforts have been made in the construction of our warehouses to counteract it. The dampness, however, of itself, be it remembered, will not cause yellow fever, if the other elements of high temperature and filth be not added.

["The Doctor then went on to speak of the manner in which this disease attacks a neighborhood, and particularly in the case of the *Ben Franklin*, which, it is alleged, carried the yellow fever to Norfolk, but which he said was not the case."]

'*It is the fault of the City Authorities if Yellow Fever invades a City!* The disease is entirely in their hands, and they may have it or not, as they wish. The "shears of fate" which is to cut the thread of their lives is formed, as I have said, of two blades. The one is high temperature, the other a high dew-point. But the rivet of these shears, without which they cannot act, is filth. The authorities can so drain the city and so thoroughly cleanse it, that one blade will be dulled, and the rivet may entirely be wanting.

'*Millions for Cure, but not a Cent for Prevention*, seems to be the motto of our city authorities. Filth is the electric spark which fires the other elements. Typhus, small-pox, yellow fever, measles, and many other diseases, as well as all intermittents, may be, in my opinion, generated without foreign importation. A study of meteorology was absolutely necessary for the safety of a city. Notwithstanding the proximity of government institutions to the infected region around Norfolk, no such observations were made, and in some places where this was supposed to be done they were made at a distance of a mile from the location where the disease raged, upon a hill, or in a healthy locality. The reports, therefore, were not the reports of the infected locality, but of a proximate healthy one, and a comparison would show a most marked difference.

'Dr. Clark hoped, if the vote was taken, it might again be taken after the discussion of the subject.

'Dr. Isaac Wood thought it unwise to vote on this question, as he doubted if the members of the Academy generally were able to vote understandingly.

'Dr. Griscom desired further light upon it before voting, and would, therefore, request Prof. David B. Reid, of Edinburgh, Scotland, whom he saw present (the distinguished chemist, well known for his scientific sanitary reports, made to the British Government; who arranged the ventilating apparatus of the New Houses of Parliament, &c.), to state to the Academy his views respecting the influence of dampness in producing disease.

'Prof. Reid, in responding, said he had seen little of the relation of dampness to yellow fever, but he had in Scotland, England, France, Russia, &c. noted many relations between moisture and disease generally. He had never in his life listened with so great interest and delight to any paper as to the erudite one of this evening. He had had much to do with ventilating old buildings in London; in draining sunken places, where the drains and air-tubes had to be carried down through the remains of the old Roman walls; ships from China; the Houses of Parliament, and especially the worst part of London, the Old Bailey. Lime, in large quantities, he had found to entirely destroy all dampness. He was not prepared to hear of the low temperature of New Orleans. He had, indeed, seen great cold in London immediately after a storm. Fever, he said, was invariably arrested by the withdrawal of moisture, to be effected in three ways: either by a high temperature drying it up, a low temperature condensing it, or by chemically withdrawing it. The difference of moisture he had personally noticed in his late residence in London, where, in the upper stories, meat would keep pure for several days, when near the ground two or three hours only would be necessary to materially change it.'

